# Prediction of Treatment Response by Thyroid Bed Uptake on Post-Ablative Whole-Body Scan in Intermediate-Risk Patients with Papillary Thyroid Cancer

**DOI:** 10.3390/diagnostics16010019

**Published:** 2025-12-20

**Authors:** Eunkyoung Choi, Yong-An Chung, Soo Jin Kwon, Jinkyoung Oh

**Affiliations:** 1 Department of Radiology, Incheon St. Mary’s Hospital, College of Medicine, The Catholic University of Korea, Seoul 06591, Republic of Korea; eet0224@gmail.com (E.C.); nucmedkr@gmail.com (Y.-A.C.); 2 Department of Radiology, Eunpyeong St. Mary’s Hospital, College of Medicine, The Catholic University of Korea, Seoul 06591, Republic of Korea; syung84@gmail.com

**Keywords:** thyroid cancer, papillary, Iodine-131, radionuclide imaging

## Abstract

**Background/Objectives**: This study aimed to evaluate the prognostic significance of thyroid bed uptake on post-ablative whole-body scan (PAWBS) in predicting treatment response in intermediate-risk papillary thyroid carcinoma (PTC) patients undergoing high-dose radioactive iodine (RAI) therapy following total thyroidectomy. **Methods**: This retrospective study included 148 intermediate-risk PTC patients who underwent high-dose RAI therapy after total thyroidectomy. PAWBS was performed 7 days post-therapy, and thyroid bed uptake was visually graded. Treatment response was assessed using stimulated thyroglobulin (sTg) levels, imaging studies, and clinical follow-up. Responses were classified into excellent, indeterminate, biochemical incomplete, or structural incomplete categories. Logistic regression analyses were conducted to identify predictors of treatment response. **Results**: Among the 148 patients, 126 (85.1%) achieved an excellent response (ER), while 22 (14.9%) showed a non-excellent response (NER), which included indeterminate, biochemical incomplete, and structural incomplete responses. Patients with NER exhibited significantly higher thyroid bed uptake on PAWBS compared to ER patients (*p* = 0.001). Multivariate analysis revealed that higher thyroid bed uptake was an independent negative prognostic factor for achieving an excellent response (*p* < 0.001), along with sTg (*p* < 0.001). **Conclusions**: The intensity of thyroid bed uptake on PAWBS independently predicts treatment response in intermediate-risk PTC patients receiving high-dose RAI therapy, with higher uptake indicating a worse prognosis.

## 1. Introduction

Thyroid cancer is one of the most common malignancies, with a rising incidence over recent decades. Papillary thyroid carcinoma (PTC) accounts for the majority of thyroid cancer and has a very favorable prognosis, with a 10-year survival rate of 85–93% [1]. Radioactive iodine (RAI) therapy is a standard treatment for PTC patients following total thyroidectomy, aimed at ablation of residual thyroid tissue and treatment of known or presumed residual or metastatic disease [2].

According to the 2015 American Thyroid Association (ATA) risk of recurrence stratification, thyroid cancer patients are categorized into low, intermediate, and high-risk groups [2,3]. High-risk thyroid cancer patients require higher doses of RAI therapy and additional treatments to improve outcomes, especially for those with distant metastasis. In contrast, low-risk patients have a low likelihood of recurrence and therefore are managed primarily with appropriate follow-up strategies, avoiding unnecessary treatments. However, managing intermediate-risk PTC patients remains challenging, as their clinical outcomes vary widely and require individualized treatment strategies to optimize prognosis.

Post-ablative whole-body scan (PAWBS) is recommended to be performed 5–10 days after RAI therapy. It plays a role in identifying lesions not detected prior to treatment and offers significant prognostic information related to treatment outcomes [4,5,6]. Although there have been reports suggesting that thyroid bed uptake in PAWBS is associated with prognosis, research in this area is still limited [5,6,7]. Furthermore, there was no information in the literature focusing on response evaluation in intermediate-risk PTC patients. Given the heterogeneity of this risk group and the need for individualized treatment approaches, assessing the prognostic significance of thyroid bed uptake on PAWBS may aid in optimizing patient management. The aim of this study was, therefore, to predict treatment response by evaluating thyroid bed uptake on PAWBS in intermediate-risk PTC patients who underwent high-dose RAI therapy after total thyroidectomy.

## 2. Materials and Methods

This study was approved by the institutional review board in the hospital. The requirement for written informed consent was waived due to the retrospective nature of the study and the use of anonymized data. We conducted a retrospective review of the medical records of PTC patients who underwent total thyroidectomy at our facility from January 2017 through December 2021. Clinicopathological data were obtained through an electronic medical record review, and the patients were classified as low, intermediate, or high risk using the 2015 ATA risk of recurrence stratification system [2]. Patients were included if they were classified as intermediate risk and had received high-dose RAI therapy after total thyroidectomy. Patients were excluded from the study based on the following criteria: (1) previous RAI therapy (low or high dose); (2) diagnosis of distant metastasis prior to receiving RAI therapy; (3) high antithyroglobulin antibody (TgAb) at the time of ablation; (4) presence of any significant liver disease; and (5) absence of available follow-up data. Pathological characteristics were evaluated in the surgical specimens obtained from the total thyroidectomy by an experienced pathologist: primary tumor size, presence of extrathyroid extension, multifocality, extranodal spread, and lymph node metastasis.

Treatment strategies were individualized based on a multi-disciplinary discussion with clinicians and nuclear medicine physicians, taking into account the clinical risk stratification for thyroid cancer. All patients received levothyroxine orally following total thyroidectomy. After withdrawal of thyroid hormone or injection of recombinant human thyroid-stimulating hormone (rh-TSH), the patients were given RAI therapy with doses ranging from 3.7 GBq to 5.55 GBq. Considering the higher iodine intake in South Korea compared with other countries, the patients were kept on a low-iodine diet for 2–3 weeks prior to I-131 ablation. For patients who received RAI therapy after thyroid hormone withdrawal, stimulated Tg (sTg) was measured in the third week of hormone withdrawal. For those who received I-131 treatment after injection of rh-TSH, sTg was measured on the third day (D3) following rh-TSH injection.

**Figure 1:**
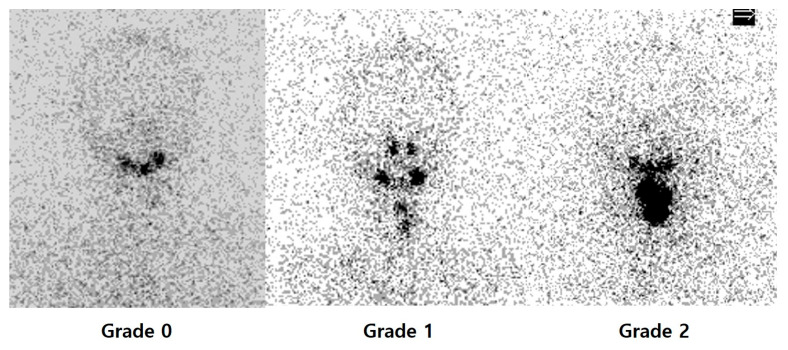
Post-ablative whole-body scan images acquired 7 days post- radioactive iodine therapy, showing thyroid bed uptake graded visually: grade 0 = no uptake; grade 1 = mild; grade 2 = moderate to intense.

**Figure 2:**
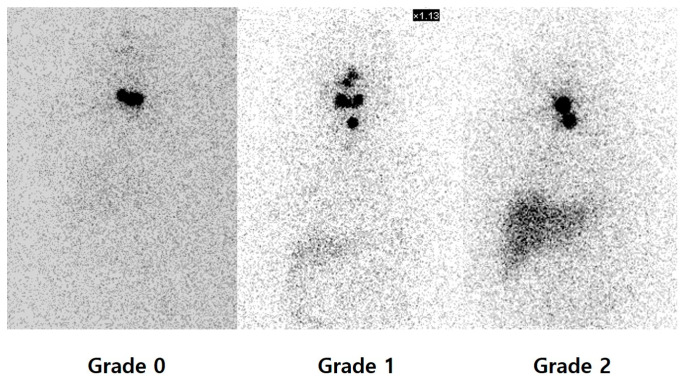
Post-ablative whole-body scan images acquired 7 days post- radioactive iodine therapy, showing hepatic uptake graded visually: grade 0 = no uptake; grade 1 = mild; grade 2 = moderate to intense.

PAWBS is performed 7 days following RAI therapy using a dual-head gamma camera equipped with high-energy collimators. The main parameter assessed was the uptake in the thyroid bed with visual analysis into 3 grades according to the following semi-quantitative system, depending on intensity: grade 0 = no uptake; grade 1 = mild; grade 2 = moderate to intense, as independently rated by two nuclear medicine physicians (Figure 1) [5,6]. Image interpretation was independently performed by two board-certified nuclear medicine physicians (J.O. and E.C.), who were blinded to the patients’ clinical information, except for body weight, which was considered due to its potential impact on photon attenuation. Discrepancies between readers were resolved by consensus. Discrepancies between readers were resolved by consensus. In cases where uptake patterns appeared atypical—such as uptake outside the thyroid bed or linear midline uptake—additional imaging (e.g., neck ultrasonography or CT) was reviewed, and final interpretation was made by consensus. In addition, diffuse liver uptake was scored by visual assessment into 3 grades in the same way (Figure 2).

Therapy response was evaluated using sTg levels, neck ultrasound findings performed by an experienced radiologist, radioiodine scans, and any additional imaging conducted during the follow-up period, which ranged from 6 to 12 months. According to the 2015 ATA guidelines, therapy responses were classified as follows: (1) excellent response: negative imaging and either suppressed Tg < 0.2 ng/mL or TSH-stimulated Tg < 1 ng/mL; (2) indeterminate response: non-specific findings on imaging studies or suppressed Tg that is detectable but <1 ng/mL or stimulated Tg between 1 and 10 ng/mL; (3) biochemical incomplete response: negative imaging and suppressed Tg ≥ 1 ng/mL or stimulated Tg ≥ 10 ng/mL or increased TgAb values; (4) structural incomplete response: structural or functional evidence of disease with any Tg level. The response to RAI therapy was categorized into excellent response (ER) and non-excellent response (NER), which includes indeterminate response, biochemical incomplete response, and structural incomplete response. We analyzed the correlation between the intensities of thyroid bed and liver uptake on PAWBS and these response categories.

Statistical analyses were performed using Stata software (version 13.0; StataCorp, College Station, TX, USA). The normality of distribution for continuous variables was assessed using the Kolmogorov–Smirnov test. Parametric variables were expressed as mean ± standard deviation, whereas non-parametric variables were presented as median with range. Categorical variables are presented as frequencies with percentages. For comparisons between groups, the chi-square test or Fisher’s exact test was used for categorical variables. Continuous variables were analyzed using Student’s *t*-test for parametric data and the Mann–Whitney U test for non-parametric data. The correlation between variables was evaluated by Spearman’s rank correlation. Variables identified as significantly affecting treatment response in univariate analyses were entered into a multivariate logistic regression model to determine the independent predictors of remission. *p*-values < 0.05 were considered statistically significant.

## 3. Results

### 3.1. Patient Characteristics

A total of 148 patients were included in this study. Of these, 35 (23.5%) were men and 113 (76.5%) were women. The mean age at diagnosis was 46 years (range, 20–84 years). Based on the treatment response, 126 patients (85.1%) were classified as ER, and 22 (14.9%) as NER. Among the 22 NER patients, 18 had a biochemical incomplete response, and 4 had an indeterminate response. Detailed clinical and pathological characteristics at the time of RAI therapy are summarized in Table 1.

### 3.2. Relationship Between Uptake on PAWBS and Therapy Response

Thyroid bed uptake on PAWBS showed a significant association with treatment response. Among the 148 patients, 10 (6.8%) showed grade 0 intensity, 86 (58.1%) showed grade 1, and 52 (35.1%) showed grade 2 intensity. In the ER group, 10 patients had grade 0, 80 had grade 1, and 36 had grade 2 intensity. In contrast, in the NER group, none had grade 0, 6 had grade 1, and 16 had grade 2 intensity. The distribution of thyroid bed uptake differed significantly between ER and NER groups (*p* = 0.001, Table 1). Liver uptake on PAWBS did not show a significant association with treatment response. Five patients (3.4%) showed grade 0 intensity, 43 (29.1%) had grade 1, and 100 (67.6%) had grade 2. In the ER group, 4 had grade 0, 38 had grade 1, and 84 had grade 2 intensity. In the NER group, 1 had grade 0, 5 had grade 1, and 16 had grade 2 intensity. The difference between the groups was not statistically significant (*p* = 0.755, Table 1).

### 3.3. Relationship Between sTg and Thyroid Bed Uptake on PAWBS

**Figure 3:**
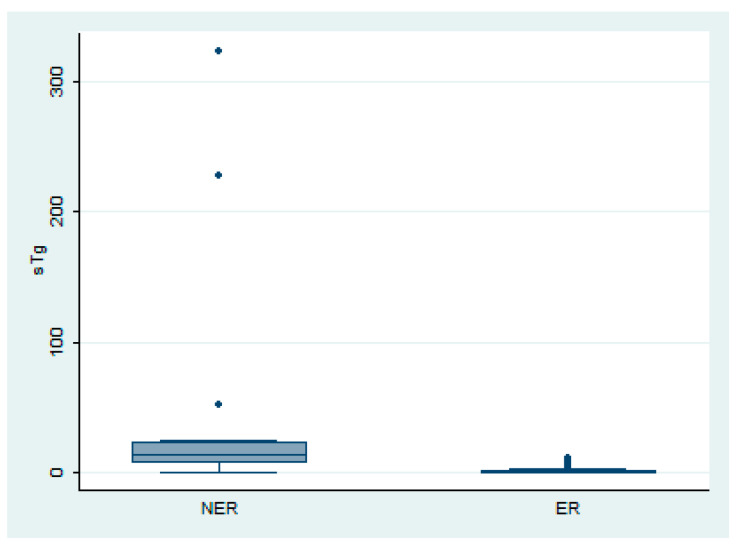
Comparison of stimulated thyroglobulin levels between the non-excellent response (NER) and excellent response (ER) groups.

The mean sTg level among all patients was 6.61 ng/mL. In the ER group, the mean sTg was significantly lower at 1.69 ng/mL (range, 0.01–37.51), compared to 36.88 ng/mL (range, 0.1–323.2) in the NER group (*p* < 0.001, Mann–Whitney U test, Figure 3). A positive correlation was observed between sTg levels and the intensity of thyroid bed uptake on PAWBS (Spearman r = 0.298, *p* < 0.001), suggesting that higher sTg levels are associated with stronger uptake on PAWBS.

### 3.4. Uni- and Multivariate Analyses

Univariate logistic regression analysis revealed that the intensity of thyroid bed uptake on PAWBS, sTg, lateral lymph node metastasis, and extranodal spread were significantly associated with therapy response (*p* < 0.001, *p* < 0.001, *p* = 0.049, and *p* = 0.005, respectively).

In the multivariate logistic regression analysis, thyroid bed uptake intensity and sTg remained significant independent predictors of therapy response (both *p* < 0.001, Table 2).

Other clinicopathological features, including primary tumor size, extrathyroidal extension, multifocality, extranodal spread, central lymph node metastasis, and lateral lymph node metastasis, showed no significant association with treatment response.

## 4. Discussion

This study demonstrated that the intensity of thyroid bed uptake on PAWBS and sTg level are significant predictors of treatment response in intermediate-risk PTC patients. These findings highlight the role of meticulous post-therapy evaluation, suggesting that patients exhibiting higher thyroid bed uptake on PAWBS and pre-RAI sTg level may benefit from closer surveillance and additional therapeutic interventions. The National Comprehensive Cancer Network (NCCN) guidelines advocate for the use of PAWBS following RAI therapy, emphasizing its utility in detecting previously unrecognized lesions and differentiating between iodine-avid and non-avid disease [8]. Expanding upon this established diagnostic utility, our findings propose that PAWBS also provides valuable prognostic information regarding therapy response, particularly within intermediate-risk patients.

Although previous studies have reported that intense thyroid bed uptake on PAWBS may serve as an independent predictor of treatment outcomes, the majority of these investigations have not specifically targeted intermediate-risk patients—a subgroup that constitutes a substantial proportion of PTC cases [5,6,9]. A significant challenge in managing this subgroup is the wide heterogeneity of clinical outcomes observed in intermediate-risk patients. This subgroup, which constitutes a substantial portion of PTC patients, requires individualized treatment and surveillance plans to optimize prognosis [10,11]. To the best of our knowledge, the present study is the first to systematically assess the prognostic significance of thyroid bed uptake intensity on PAWBS exclusively in intermediate-risk PTC patients. The degree of thyroid bed uptake may reflect the burden of remnant tumor or residual thyroid tissue, both of which have been identified as factors associated with unsuccessful outcomes following adjuvant therapy [6,12]. This was further supported by our analysis demonstrating a significant association between thyroid bed uptake intensity and sTg level.

sTg serves as a surrogate marker for the burden of remnant thyroid tissue, which is closely associated with treatment response [13]. A low sTg level at the time of remnant ablation may indicate a favorable prognosis. Similarly, thyroid bed uptake observed on PAWBS may indirectly reflect sTg levels and, therefore, function as a predictor of therapeutic outcomes. Integrating sTg with thyroid bed uptake findings on PAWBS may improve the accuracy of treatment response prediction and support more individualized therapeutic strategies for intermediate-risk PTC patients.

In this study, univariate analysis revealed that among various clinicopathological parameters, lateral lymph node metastasis and extranodal spread were significantly associated with a non-excellent treatment response. Extranodal spread, the extension of tumor cells beyond the lymph node capsule into surrounding tissue, is associated with a significantly higher risk of recurrence and compromised disease-specific survival [14,15]. This underscores the importance of a thorough pathological review of surgical specimens, as the presence of extranodal spread can have a profound impact on a patient’s long-term prognosis. Similarly, lateral lymph node metastasis has been repeatedly linked to unfavorable therapeutic outcomes and higher rates of locoregional recurrence or distant metastasis, supporting its role as a key prognostic marker in PTC [16,17].

Kim et al. demonstrated that both thyroid bed uptake and liver uptake on PAWBS were significant prognostic factors for treatment response in differentiated thyroid cancer patients [6]. A notable discrepancy in our study’s findings is the lack of a significant correlation between liver uptake on PAWBS and treatment response, contrasting with a previous study by Kim et al. This contrast may be attributed to differences in the patient populations included in each study. Kim et al. included advanced thyroid cancer patients with substantial residual thyroid tissue or metastatic disease, whereas our study excluded patients with overt thyroid cancer tissue or distant metastases prior to RAI therapy. Regarding the prognostic implications of liver uptake, previous research has suggested that diffuse hepatic visualization on PAWBS may reflect the degree of thyroid tissue destruction or the presence of functioning metastases [5,18]. It has been proposed that tissue binding of I-131-labeled iodoproteins contributes to diffuse hepatic uptake on PAWBS. Although liver uptake has been associated with poorer treatment responses in certain contexts, our findings suggest that, in intermediate-risk PTC patients without significant residual disease, liver uptake intensity may not serve as a reliable predictor of treatment response.

There are several limitations in this study. First, because of the retrospective nature of the study design, selection bias may have been introduced. A larger, multicenter cohort would strengthen the validity and applicability of the results across diverse populations and clinical settings, and allow for a more robust evaluation of recurrence and survival outcomes with longer follow-up. Second, the visual grading of thyroid bed uptake was performed on planar scans, which inherently lack the anatomical resolution and quantitative capability of hybrid imaging modalities such as single-photon emission computed tomography/computed tomography (SPECT/CT). Future studies employing quantitative SPECT/CT analysis could enhance the objectivity and reproducibility of thyroid bed uptake evaluation. Third, patient classification and treatment response categories were based on the 2015 ATA guidelines [2]. Future studies applying the 2025 criteria will be needed to ensure consistency with current clinical practice [19].

In conclusion, thyroid bed uptake on post-ablative whole-body scan is an independent negative predictor of treatment response in intermediate-risk PTC patients. Along with elevated sTg levels, increased thyroid bed uptake may reflect the extent of remnant tumor or residual thyroid tissue and help identify patients who require closer surveillance and tailored management following high-dose RAI therapy. These findings support incorporating post-therapy scan findings into treatment planning and follow-up strategies to improve patient outcomes.

## Figures and Tables

**Table 1:** Patient characteristics (*N* = 148).

Characteristics	Excellent Response (*n* = 126)	Non-Excellent Response (*n* = 22)	*p* Values
Sex			
Female	99 (78.6%)	14 (63.6%)	0.212
Male	27 (21.4%)	8 (36.4%)	
Age (mean ± SD)	45.6 ± 13.1	49.9 ± 14.3	0.159
Thyroid bed uptake on PAWBS			*p* < 0.001
Grade 0	10 (7.9%)	0	
Grade 1	80 (63.5%)	6 (27.3%)	
Grade 2	36 (28.6%)	16 (72.7%)	
Liver uptake on PAWBS			0.755
Grade 0	4 (3.2%)	1 (4.6%)	
Grade 1	38 (30.1%)	5 (22.7%)	
Grade 2	84 (66.7%)	16 (72.7%)	
Initial sTg (ng/mL, median, range)	0.34 (0.01–10.5)	13.64 (0.3–323.2)	*p* < 0.001
Primary tumor size	1.2 (0.3–6.3)	1.9 (0.3–4.5)	0.105
Presence of extrathyroid extension	97 (77%)	17 (77.3%)	1.0
Multifocality	55 (43.7%)	9 (40.9%)	0.995
Extranodal spread	18 (14.3%)	9 (40.9%)	0.007
Central lymph node metastasis	104 (82.5%)	18 (81.8%)	1.0
Lateral lymph node metastasis	18 (14.3%)	7 (31.8%)	0.086

PAWBS, post-ablative whole-body scan; sTg, stimulated serum thyroglobulin.

**Table 2:** Multivariate analysis of clinicopathologic factors associated with treatment response.

	Coefficient	Standard Error	Odds Ratio (95% Confidence Interval)	*p* Value
Thyroid bed uptake on PAWBS	−0.1499	0.0453	0.861 (0.787–0.941)	*p* < 0.001
Stimulated thyroglobulin	−0.0035	0.0007	0.996	*p* < 0.001
Lateral lymph node metastasis	−0.128	0.0691	0.88 (0.768–1.009)	0.065
Extranodal spread	−0.0427	0.0611	0.958 (0.849–1.081)	0.486

## Data Availability

The data supporting the findings of this study are available from the corresponding author (J.O.) upon request. The data are not publicly available due to privacy and legal reasons.

## Abbreviations

The following abbreviations are used in this manuscript:

PTC	Papillary thyroid carcinoma
RAI	Radioactive iodine
ATA	American Thyroid Association
PAWBS	Post-ablative whole-body scan
rh-TSH	Recombinant human thyroid-stimulating hormone
sTg	Stimulated Tg
ER	Excellent response
NER	Non-excellent response
NCCN	National Comprehensive Cancer Network
SPECT/CT	Single-photon emission computed tomography/computed tomography
